# Induction of apoptosis and autosis in cardiomyocytes by the combination of homocysteine and copper via NOX-mediated p62 expression

**DOI:** 10.1038/s41420-022-00870-4

**Published:** 2022-02-21

**Authors:** Ran Yin, Huan Wang, Chun Li, Lulu Wang, Songqing Lai, Xianhe Yang, Daojun Hong, Wan Zhang

**Affiliations:** 1 Department of Cardiology, The First Affiliated Hospital of Nanchang University, 330006 Nanchang, Jiangxi P. R. China; 2 Department of Neurology, The First Affiliated Hospital of Nanchang University, 330006 Nanchang, Jiangxi P. R. China; 3 Department of Ultrasound, The First Affiliated Hospital of Nanchang University, 330006 Nanchang, Jiangxi P. R. China; 4 Institute of Cardiovascular Diseases, Jiangxi Academy of Clinical Medical Sciences, The First Affiliated Hospital of Nanchang University, 330006 Nanchang, Jiangxi P. R. China; 5 Medical Innovation Center, The First Affiliated Hospital of Nanchang University, 330006 Nanchang, Jiangxi P. R. China; 6 Department of Thoracic Surgery, The First Affiliated Hospital of Nanchang University, 330052 Nanchang, Jiangxi P. R. China; 7 Jiangxi Institute of Respiratory Disease, 330052 Nanchang, Jiangxi P. R. China

**Keywords:** Cardiovascular diseases, Macroautophagy, Metals

## Abstract

High levels of homocysteine (Hcy) associated with cardiovascular events are accompanied by increased copper (Cu) concentrations in the blood. Hcy has been shown to promote endothelial dysfunction, whereas the effect of Hcy on cardiomyocytes and the role of Cu in the pathogenesis remain less understood. In the present study, it is demonstrated that the combination of Hcy and Cu^2+^-induced apoptosis and autosis of cardiomyocytes simultaneously, and thus led to cardiac dysfunction in hyperhomocysteinemic rats. These effects were associated with p22^phox^ activation and NADPH oxidase (NOX)-mediated p62 upregulation. Inhibition of the expression of p22^phox^ or p62 in cardiomyocytes significantly attenuated Hcy and Cu^2+^-mediated reactive oxygen species (ROS) generation and cell death. Furthermore, interrupting the NOX–p62 axis prevented diastolic dysfunction in hyperhomocysteinemic rats (HcyR). These findings establish that the induction of apoptosis and autosis of cardiomyocytes through stimulating the NOX–p62-signaling pathway constitutes a novel mechanism of Hcy and Cu-induced cardiac dysfunction.

## Introduction

An elevated level of homocysteine (Hcy) in blood, denoted hyperhomocysteinemia, is emerging as a strong risk factor for the development of atherosclerotic vascular disease [1]. Interestingly, later studies observed a concomitant increase in blood Hcy and copper (Cu) in association with vascular dysfunction [2]. Clinical data and animal studies indicate that Hcy may also have adverse effects on the myocardium, which might due to reactive oxygen species (ROS)-mediated apoptosis in cardiomyocytes [3]. However, the precise mechanism of Hcy-mediated myocardium dysfunction and whether it is associated with Cu remain largely unknown.

Autophagy is a cytoprotective mechanism, whereas over-activated autophagy can leads to cell death, named autophagic cell death [4]. Recently, a novel form of Na^+^, K^+^-ATPase-dependent autophagic cell death, termed autosis, is identified, which is characterized by the disappearance of endoplasmic reticulum and focal swelling of the perinuclear space [5]. High levels of cellular autophagy, as occurs with autophagy-inducing peptide treatment, starvation, or in vivo during certain types of ischemia, can trigger autosis [5, 6]. In the myocardium, basal autophagy is a homeostatic mechanism for the maintenance of normal cardiac function and morphology. However, the stress-activated autophagy in cardiac disorders is more complicated, which dependents on the context and the disease studied. It has been suggested that the autophagy induced by acute pathological insults is protective whereas sustained autophagy in the chronic phase of cardiac diseases can be either beneficial or detrimental, depending on the nature of the stress [7], which is still controversial and needs further exploration.

Apoptosis could occur with autophagy simultaneously [8]. Emerging evidence suggests interactions among the crucial proteins of autophagy and apoptosis, which underlie the crosstalk between them [9]. Caspases are key proteins in the extrinsic apoptotic pathway that could participate in regulating the crosstalk between autophagy and apoptosis in cancer and neurodegeneration [10]. On one hand, activated caspases could break down the autophagic proteins including beclin 1, p62, and Atg7, and thus inhibit the autophagic process [11, 12]. Or, caspases activated by pro-apoptosis stimuli could cleave and transform pro-autophagic proteins into pro-apoptotic proteins to initiate apoptotic cell death [13]. On the other hand, autophagy can modulate the caspases and thus affect apoptotic cascades [14]. However, the crosstalk of autophagy and apoptosis in cardiovascular diseases is still under debate and has great research potential.

p62, also known as sequestosome 1 (SQSTM1), is a well-known significant regulator of selective autophagy [15]. At physiological conditions, basal autophagy maintains the p62 at relatively low levels. However, the rise in p62 levels due to increased transcription or decreased autophagy has been observed in various diseases [16–18]. p62 is found to be a multifunctional molecule in cancer that either inhibits or induces cancer cell death [18]. While it has been established that autophagy regulates the levels of p62, there is strong evidence of the correlation between p62 and apoptosis. Studies have demonstrated that p62 acts as a signaling hub to recruit and oligomerize important signaling molecules to control cancer cell survival and apoptosis [19], suggesting that p62 might play a critical role in both autophagy and apoptosis.

Reactive oxygen species (ROS) may serve as an important intermediate factor in the relationship between Hcy and cardiovascular disease (CVD) [20]. NADPH oxidase (NOX) is one of the major enzymatic sources of ROS in the cardiovascular system [21]. Due to the ability to regulate redox signaling pathways, ROS is shown to mediate apoptosis and/or autophagy in diverse pathological conditions [22]. However, the mode of activation and the potential role of ROS in various diseases remained incompletely understood. Especially, the correlation of ROS, autophagy and apoptosis induced by Hcy in association with Cu in CVD is still undefined.

In brief, the aims of this study are to examine the combinative effect of Hcy and Cu against the survival and function of cardiomyocytes, and to investigate the molecular mechanism involved. Our results indicated that apoptosis and autosis coexist in cardiomyocytes in the presence of Hcy and Cu, and a possible mechanism is the p62-dependent caspase activation and autosis triggered by the NOX-originated ROS. Characterizing the manner by which Hcy and Cu induce cardiomyocyte damage will complement the well-established vascular effects of Hcy and provide a better understanding of the association of Hcy and CVD.

## Results

### The combination of Hcy and Cu^2+^ induces both apoptosis and autophagy in cardiomyocytes

**Fig. 1:**
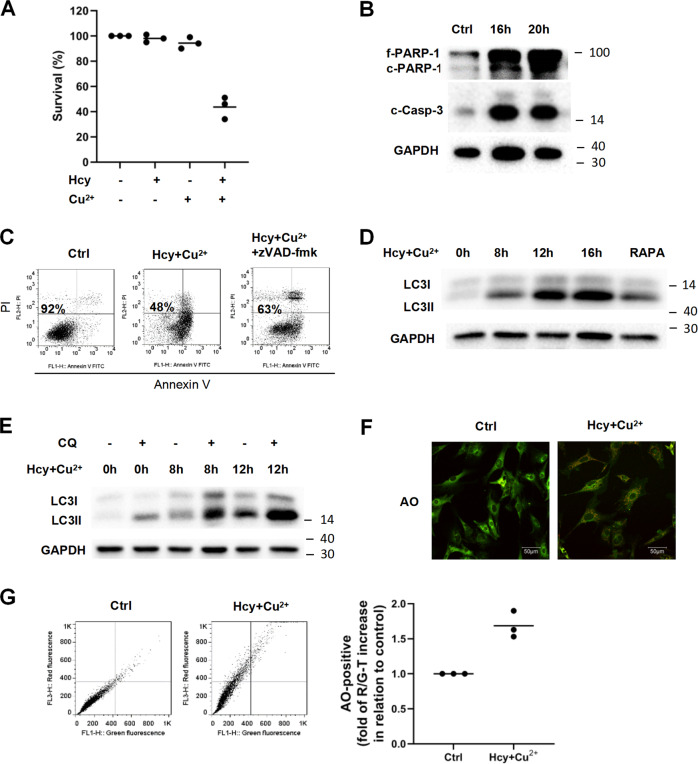
Hcy and Cu^2+^ induce both the caspase-3-dependent apoptosis and autophagy in neonatal cardiomyocytes. **A** Effect of Hcy and Cu^2+^ on cardiomyocyte viability. The cells were incubated with 800 μM Hcy and 20 μM CuCl_2_ alone or in combination for 24 h. Cell viability was analyzed by LDH release assay. Each bar represents the mean of three separate experiments, each measured in triplicate. **B** Expressions of full-length PARP-1 (f-PARP-1), cleaved PARP-1 (c-PARP-1), and cleaved caspase-3 (c-Casp-3) in cardiomyocytes with Hcy and Cu^2+^ incubation at given time points, as assessed by western blot analysis. The representative western blot results are shown, with GAPDH expression as an internal control. **C** Effect of zVAD-fmk on cardiomyocyte viability with Hcy and Cu^2+^ for 24 h. The cells were preincubated with 800 μM Hcy and 20 μM CuCl_2_ for 24 h before 20 μM zVAD-fmk was added. Cell viability was measured using annexin V/PI double staining. Representative dot plots of a cardiomyocyte sample are shown, with numbers indicating the percentage of viable cells (annexin V/PI double negative). **D** Expressions of LC3 in cardiomyocytes with Hcy and Cu^2+^ incubation at given time points, assessed by western blot analysis. The representative western blot results are shown, with GAPDH expression as an internal control. **E** Expressions of LC3 in cardiomyocytes with Hcy and Cu^2+^ incubation with or without CQ at given time points, assessed by western blot analysis. The representative western blot results are shown, with GAPDH expression as an internal control. **F** Representative images of acridine orange-stained cardiomyocytes with Hcy and Cu^2+^ incubation for 12 h. **G** Quantification of acridine orange staining using flow cytometry. Representative dot plots of three separate experiments are shown (left panel). The bar (right panel) represents the mean of three separate experiments, each measured the proportion of the events above the threshold with R/GFIR-T. All the experiments above were performed three times. Ctrl control, f-PARP-1 full-length PARP-1, c-PARP-1 cleaved PARP-1, c-Casp-3 cleaved caspase-3, CQ Chloroquine, RAPA rapamycin, R/GFIR red-to-green fluorescence intensity ratio.

As shown in Fig. 1A, the combination of Hcy and Cu^2+^ significantly reduced the survival rate of neonatal cardiomyocytes, whereas Hcy or Cu^2+^ alone had limited toxicity, indicating that Hcy induces cardiomyocyte cell death only in the presence of Cu^2+^. We observed efficient processing of caspase-3 and its substrate PARP-1 in cardiomyocytes with Hcy and Cu^2+^ treatment (Fig. 1B). However, the apoptosis in cardiomyocyte cell line H9c2 was only partially rescued by pan-caspase inhibitor zVAD-fmk (Fig. 1C), indicating that both caspase-dependent and caspase-independent cell death are involved in Hcy and Cu^2+^-induced cytotoxicity. Further analysis showed that the protein level of LC3-II increased in a time-dependent manner in neonatal cardiomyocytes treated with Hcy and Cu^2+^, which was similar to rapamycin, used as a positive control for autophagy induction (Fig. 1D). Meanwhile, “autophagic flux” assay using lysosomotropic reagent chloroquine (CQ) showed a further increase in the levels of LC3-II in cardiomyocytes, indicating that autophagic flux is increased by the incubation with Hcy and Cu^2+^ (Fig. 1E). Moreover, we also identified the development of acidic vesicular organelles (AVOs), a characteristic of the late autophagic stage, in the cytoplasm of cardiomyocytes by acridine orange (AO) staining (Fig. 1F). To exclude the nonspecific effect of AO for autophagy detection, we performed an established ratiometric analysis of AO staining followed by flow cytometry [23]. As shown in Fig. 1G, the combination of Hcy and Cu^2+^ increased the red-to-green fluorescence intensity ratio (R/GFIR), indicating the development of AVOs. Overall, the results above indicate that the combination of Hcy and Cu^2+^ induces both apoptosis and autophagy in cardiomyocytes simultaneously.

### The combination of Hcy and Cu^2+^ induces p62-depedent autosis

**Fig. 2:**
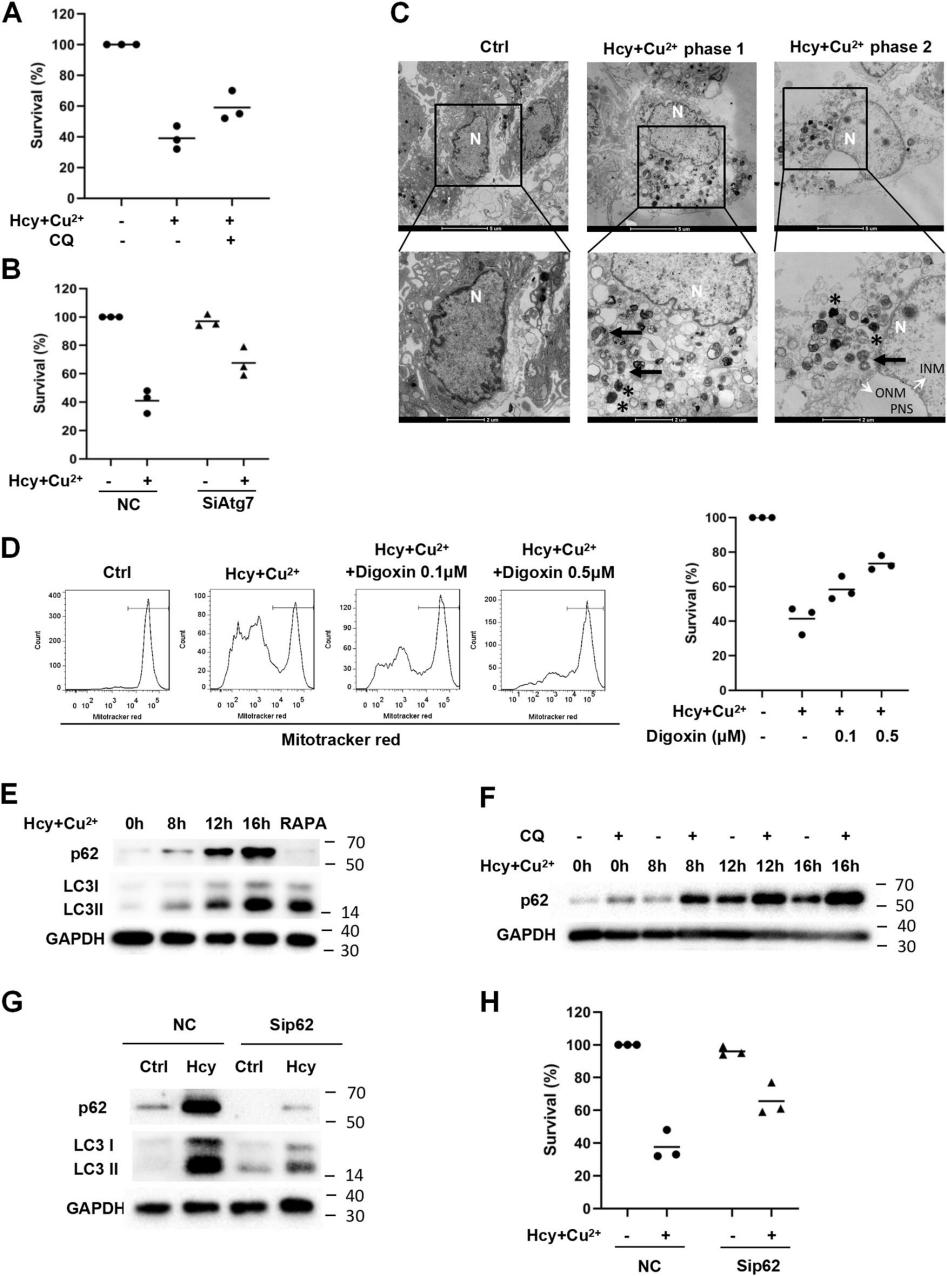
The combination of Hcy and Cu^2+^ induces p62-depedent autosis. **A** Effect of CQ on cardiomyocyte viability with Hcy and Cu^2+^ for 24 h. The cells were pre-treated with 10 μM CQ 1 h before 800 μM Hcy and 20 μM CuCl_2_ was added. Cell viability was analyzed by LDH release assay. Each bar represents the mean of three separate experiments, each measured in triplicate. **B** Reduced viability of H9c2 cells with Hcy and Cu^2+^ by Atg7 knockdown. H9c2 cells infected with Atg7 siRNA or non-targeting siRNA control (NC) was treated with Hcy and CuCl_2_ for 24 h. Cell viability was analyzed by LDH release assay. Each bar represents the mean of three separate experiments, each measured in triplicate. **C** Ultrastructure of neonatal cardiomyocytes treated with or without Hcy and Cu^2+^ for 12 h (phase 1) and 20 h (phase 2). The representative transmission electron microscopic (TEM) images are shown. Lower panels: Details of autosis. Note the swollen perinuclear space (PNS), outer nuclear membrane (ONM, white arrow) and inner nuclear membrane (INM, white arrow) in phase 2 neonatal cardiomyocyte, and the presence of early (black arrows) and late (black star) autophagosomes in Hcy and Cu^2+^-treated cells but not control ones. N nucleus. **D** The effect of digoxin on cardiomyocyte viability with Hcy and Cu^2+^ for 24 h. H9c2 cells were pre-treated with 0.1 or 0.5 μM digoxin 1 h before 800 μM Hcy and 20 μM CuCl_2_ was added. Cell death was determined by the loss of mitochondrial membrane potential detected by flow cytometry analysis (left), or assessed by the measurement of lactate dehydrogenase (LDH), released from dead cells (right). In the left panel, the numbers indicate the gating of a subpopulation of survival cells. Representative histograms of three separate experiments are shown. In the right panel, each bar represents the mean of three separate experiments, each measured in triplicate. **E** Expressions of p62 and LC3 in cardiomyocytes with Hcy and Cu^2+^ incubation at given time points, assessed by western blot analysis. The representative western blot results are shown, with GAPDH expression as an internal control and rapamycin (RAPA) as positive control. **F** Expressions of p62 in cardiomyocytes with Hcy and Cu^2+^ incubation with or without CQ at given time points, assessed by western blot analysis. The representative western blot results are shown, with GAPDH expression as an internal control. **G** Effect of p62 knockdown on LC3 expression in H9c2 cells with Hcy and Cu^2+^ incubation, assessed by western blot analysis. H9c2 cells infected with p62 siRNA or non-targeting siRNA control (NC) was treated with Hcy and CuCl_2_ for 16 h. The representative western blot results are shown, with GAPDH expression as an internal control. **H** Effect of p62 knockdown on H9c2 cell viability with Hcy and Cu^2+^ incubation. The cells were transfected with p62 siRNA or non-targeting siRNA control (NC), followed by Hcy and CuCl_2_ treatment for another 24 h. Cell viability was analyzed by LDH release assay. Each bar represents the mean of three separate experiments, each measured in triplicate. All the experiments above were performed three times. Ctrl, CQ, RAPA: see Fig. 1.

We next investigated the role of autophagy in Hcy and Cu^2+^-induced cell death. As shown in Fig. 2A, inhibition of autophagy by CQ prevented Hcy and Cu^2+^-mediated loss of cell viability, suggesting that Hcy and Cu^2+^-induced cell death is partially due to autophagy. To avoid the non-specific effect of CQ, cardiomyocyte cell line H9c2 was transfected with Atg7 siRNA, which significantly attenuated autophagic activity (Fig. S1). As shown in Fig. 2B, siAtg7 significantly reduced Hcy and Cu^2+^-mediated cardiomyocyte cell death, confirming that Hcy and Cu^2+^ can lead to autophagic cell death. High levels of autophagy might result in autosis, which has unique morphological changes [5]. We performed ultrastructural analyses to characterize the morphology of cardiomyocyte cell death. Figure 2C shows the early (phase 1) and late phase (phase 2) of cell death. Numerous membranous vacuoles and autophagosomes appeared in the phase 1 of neonatal cardiomyocyte. Notably, the phase 2 of the cells, characterized by an abrupt phase of final collapse and cell death, demonstrated the unique morphological features of autosis [5], including the concave nucleus, swollen prinuclear space (PNS), focal separation of the inner (INM) and outer nuclear membrane (ONM), and abundant lysosomes and autophagosomes. Moreover, since autosis is a form of cell death that specifically relying on the Na^+^/K^+^-ATPase [5, 6]. We have tested the effect of digoxin, one of the cardiac glycosides that inhibit Na^+^, K^+^-ATPase, on cardiomyocyte cell death in the presence of Hcy and Cu^2+^. Figure 2D shows that digoxin greatly rescued Hcy and Cu^2+^-induced cardiomyocyte cell death, suggesting that Hcy and Cu^2+^-mediated autophagic cell death is autosis. We further explored the mechanisms underlying the autosis induced by Hcy and Cu^2+^ in cardiomyocytes. During autophagy, SQSTM1 encodes the cargo adaptor protein p62 that interacts with autophagic substrates and delivers them to autophagosomes for degradation, and p62 is itself degraded and a corresponding decrease in p62 levels is usually observed [24]. Interestingly, in contrast to rapamycin, Hcy and Cu^2+^-mediated induction of autophagy was accompanied by an increased expression of p62, which correlated with the degrees of LC3 conversion (Fig. 2E). Administration of CQ to inhibit lysosomal activity enhanced Hcy and Cu^2+^-induced increase in the p62 expression, indicating the accumulation of p62 is not due to the impairment in the autophagic flux (Fig. 2F). Further analysis showed that p62 knockdown resulted in a total blockage of the LC3-II accumulation induced by Hcy and Cu^2+^ treatment (Fig. 2G). Importantly, the p62 inhibition restored cell viability in the presence of Hcy and Cu^2+^ (Fig. 2H), indicating that the p62 is required for Hcy and Cu^2+^-mediated autosis.

### p62 accumulation mediates Hcy and Cu^2+^-induced caspase cleavage and apoptosis

**Fig. 3:**
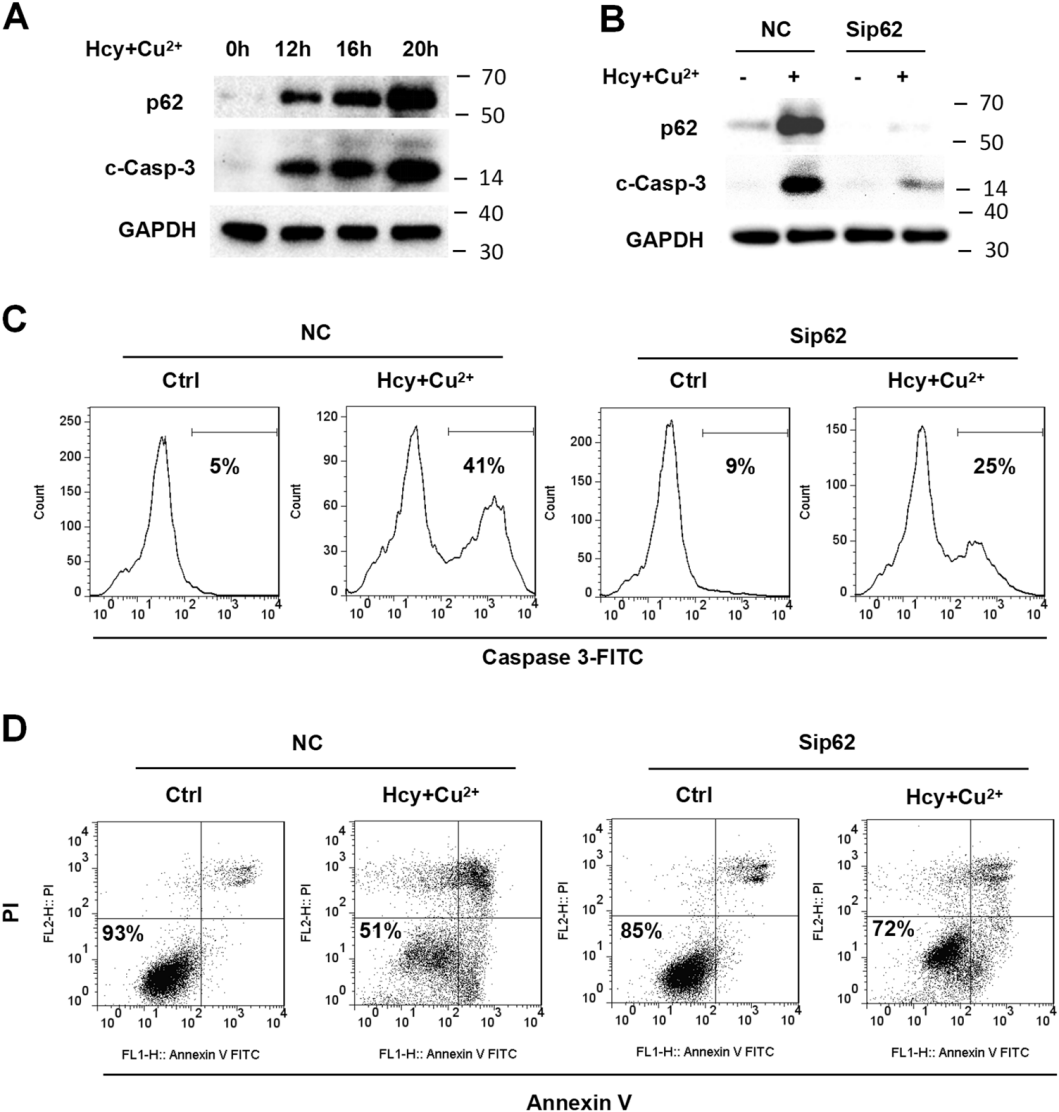
p62 accumulation is required for Hcy and Cu^2+^-induced caspase-3 cleavage and apoptosis. **A** Expression of p62 and cleaved caspase-3 (c-Casp-3) in cardiomyocytes with Hcy and Cu^2+^ incubation at given time points, assessed by western blot analysis. The representative western blot results are shown, with GAPDH expression as an internal control. **B** Effect of p62 knockdown on the cleavage of caspase-3 in H9c2 cells with Hcy and Cu^2+^ incubation, assessed by western blot analysis. H9c2 cells infected with p62 siRNA or non-targeting siRNA control (NC) was treated with Hcy and CuCl_2_ for 20 h. The representative western blot results are shown, with GAPDH expression as an internal control. **C** Effect of p62 knockdown on the activity of caspase-3 in H9c2 cells with Hcy and Cu^2+^ incubation. H9c2 cells infected with p62 siRNA or non-targeting siRNA control (NC) was treated with Hcy and CuCl_2_ for 20 h. Caspase-3 activation was detected by flow cytometry analysis. The numbers indicate the gating of a subpopulation of cells with positive caspase-3 activation. Representative histograms of three separate experiments are shown. **D** Effect of p62 knockdown on H9c2 viability with Hcy and Cu^2+^ incubation. H9c2 cells infected with p62 siRNA or non-targeting siRNA control (NC) was treated with Hcy and CuCl_2_ for 24 h. Cell viability was measured using annexin V/PI double staining. Representative dot plots of a sample are shown, with numbers indicating the percentage of viable cells (annexin V/PI double negative). All the experiments above were performed three times. Ctrl control, NC non-targeting siRNA control, Sip62 p62 siRNA, c-Casp-3 cleaved caspase-3.

A number of recent publications have shown that p62 is a multifunctional scaffolding protein that interacts with a variety of proteins to regulate diverse processes including other forms of cell death like apoptosis. We found the trend of p62 level in Hcy and Cu^2+^-treated cells correlated with the degrees of caspase-3 cleavage (Fig. 3A). To address if the p62 functions in apoptosis, we examined the effect of p62 knockdown on caspase-3 cleavage. The results in Fig. 3B shows that the suppression of p62 by siRNA in H9c2 cells significantly attenuated the caspase-3 cleavage induced by the treatment of Hcy and Cu^2+^. Consistently, flow cytometry showed that the p62 knockdown reduced Hcy and Cu^2+^-induced caspase-3 activity (Fig. 3C). To further examine the ability of p62 to regulate apoptosis, we determined whether the p62 knockdown influenced Hcy and Cu^2+^-mediated apoptosis. As shown in Fig. 3D, suppression of p62 by siRNA significantly diminished apoptosis induced by Hcy and Cu^2+^. These results indicate that p62 up-regulation can enhance Hcy and Cu^2+^-mediated caspase-3 cleavage and the following apoptosis.

### NOX-originated ROS induces p62 expression followed by the crosstalk of autophagic and apoptotic cell death in cardiomyocytes with Hcy and Cu^2+^ treatment

**Fig. 4:**
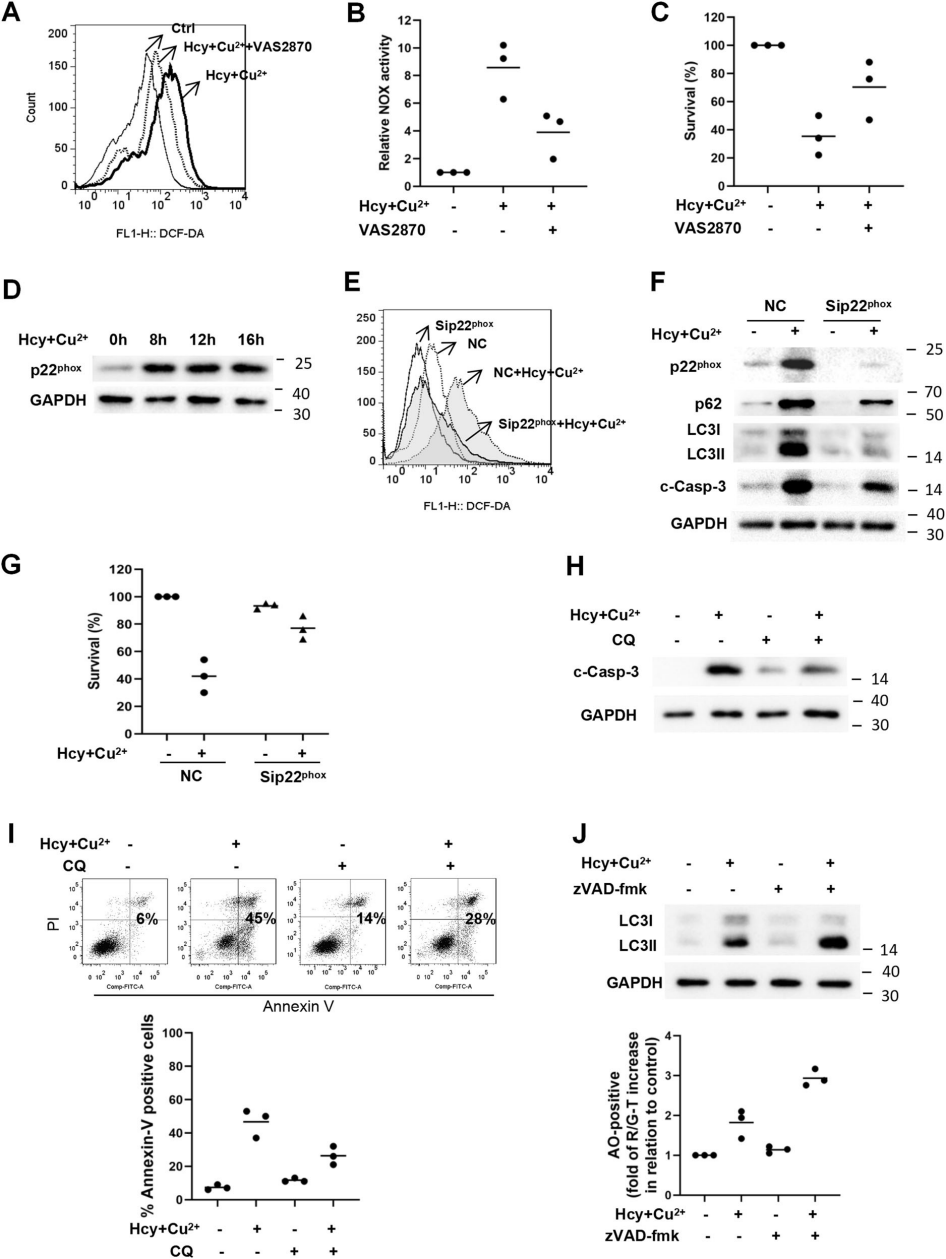
Hcy and Cu^2+^-mediated p22^phox^ upregulation and p62 accumulation induce autophagic and apoptotic cell death in cardiomyocyte. **A** Determination of cellular ROS in cardiomyocytes with Hcy and Cu^2+^ in the presence or absence VAS2870 for 8 h, detected by flow cytometry analysis. The cells were pre-treated with 5 μM VAS2870 1 h before 800 μM Hcy and 20 μM CuCl_2_ was added. Representative histograms of three separate experiments are shown. **B** NOX activity in cardiomyocytes with Hcy and Cu^2+^ in the presence or absence of VAS2870 for 8 h. The cells were pre-treated with 5 μM VAS2870 1 h before 800 μM Hcy and 20 μM CuCl_2_ was added. NOX activity was determined by chemiluminescence assays. Each bar represents the mean of three separate experiments. **C** Effect of VAS2870 on cardiomyocyte viability with Hcy and Cu^2+^ for 24 h. The cells were pre-treated with 5 μM VAS2870 1 h before 800 μM Hcy and 20 μM CuCl_2_ was added. Cell viability was analyzed by LDH release assay. Each bar represents the mean of three separate experiments, each measured in triplicate. **D** Expression of p22^phox^ in cardiomyocytes with Hcy and Cu^2+^ incubation at given time points, assessed by western blot analysis. The representative western blot results are shown, with GAPDH expression as an internal control. **E** Effect of p22^phox^ knockdown on cellular ROS in cardiomyocytes with Hcy and Cu^2+^ incubation. H9c2 cells infected with p22^phox^ siRNA or non-targeting siRNA control (NC) was treated with Hcy and CuCl_2_ for 8 h. Cellular ROS was detected by flow cytometry analysis. Representative histograms of three separate experiments are shown. **F** Effect of p22^phox^ knockdown on the expression of p62, LC3, and cleaved caspase-3 (c-Casp-3) in H9c2 cells with Hcy and Cu^2+^ incubation, assessed by western blot analysis. H9c2 cells infected with p22^phox^ siRNA or non-targeting siRNA control (NC) was treated with Hcy and CuCl_2_ for 16 h. The representative western blot results are shown, with GAPDH expression as an internal control. **G** Effect of p22^phox^ knockdown on H9c2 viability with Hcy and Cu^2+^ incubation. H9c2 cells infected with p22^phox^ siRNA or non-targeting siRNA control (NC) was treated with Hcy and CuCl_2_ for 24 h. Cell viability was analyzed by LDH release assay. Each bar represents the mean of three separate experiments, each measured in triplicate. NC non-targeting siRNA control, c-Casp-3 cleaved caspase-3. **H** Effect of CQ on caspase-3 cleavage of H9c2 with Hcy and Cu^2+^ incubation. The cells were pre-treated with 10 μM CQ 1 h before 800 μM Hcy and 20 μM CuCl_2_ was added. The representative western blot results of the expression of cleaved caspase-3 (c-Casp-3) are shown, with GAPDH expression as an internal control. **I** Effect of CQ on the apoptotic cell death of H9c2 cells with Hcy and Cu^2+^ incubation. Cell viability was measured using annexin V/PI double staining. Representative dot plots of a cardiomyocyte sample are shown, with numbers indicating the percentage of dead cells (annexin V positive, upper panel). The data are summarized in the graph in the lower panel. **J** Effect of zVAD-fmk on the autophagy of H9c2 cells with Hcy and Cu^2+^ incubation, assessed by western blot analysis (upper panel) and acridine orange staining (lower panel). The cells were pre-treated with 20 μM zVAD-fmk 1 h before 800 μM Hcy and 20 μM CuCl_2_ was added. The bar in the lower panel represents the mean of three separate experiments, each measured the proportion of the events above the threshold with R/GFIR-T. All the experiments above were performed three times. NOX NADPH oxidase, Sip22^phox^ p22^phox^ siRNA, Ctrl, NC, CQ, c-Casp-3, R/GFIR: see Figs. 1 and 3.

Since NOX is involved in the pathogenesis of many vascular diseases, we investigated the role of NOX in Hcy and Cu^2+^-mediated cardiomyocyte cell death. As shown in Fig. 4A, cellular ROS in neonatal cardiomyocytes was increased after cultured with Hcy and Cu^2+^, which was greatly diminished by the pre-incubation of NOX inhibitor VAS2870. Meanwhile, Hcy and Cu^2+^ increased NOX activity of neonatal cardiomyocytes by almost 8-fold, which was largely inhibited by VAS2870 (Fig. 4B), indicating that the ROS was originated from NOX. Further analysis demonstrated that VAS2870 promoted the overall survival rate of neonatal cardiomyocytes after cultured with Hcy and Cu^2+^ (Fig. 4C). Collectively, these results indicate that NOX is the main source of ROS in cardiomyocytes in the presence of Hcy and Cu^2+^. As p22^phox^ is a vital subunit for the functional NO*X* to generate ROS, we investigated the expression of p22^phox^ by immunohistochemistry and western blot. As shown in Fig. 4D, Hcy and Cu^2+^ treatment triggered the upregulation of p22^phox^ expression in cardiomyocytes in a time-dependent manner. By targeting the p22^phox^ with siRNA to reduce NOX activity (Fig. S2), we found that knockdown of p22^phox^ reduced endogenous ROS levels, and further diminished Hcy and Cu^2+^-stimulated ROS in cardiomyocytes (Fig. 4E). Importantly, knockdown of p22^phox^ largely attenuated the accumulation of LC3-II and p62, prevented the cleavage of caspase-3, and restored cell viability of cardiomyocytes in the presence of Hcy and Cu^2+^ (Fig. 4F, G). These data suggest that p22^phox^ upregulation-induced ROS generation promotes autophagic and apoptotic signaling, and thus leads to Hcy and Cu^2+^-mediated cardiomyocyte cell death. Meanwhile, the relationship between apoptosis and autophagy was also investigated in our study. As shown in Fig. 4H, I, CQ could prevent the cleavage of caspase-3 and partly block Hcy and Cu^2+^-induced apoptosis; on the other hand, zVAD-fmk further increased autophagy (Fig. 4J), indicating that there is crosstalk between Hcy and Cu^2+^-induced apoptosis and autophagy of cardiomyocyte. Notably, we found that the expression of Bcl-2 was decreased, whereas neither change in expression nor cleavage of beclin 1 were observed (Fig. S3), indicate that the degradation of Bcl-2, but not beclin 1, might be involved in the physiologic link between autophagic and apoptotic signaling in Hcy and Cu^2+^-treated cardiomyocyte.

### NOX–p62 axis-mediated apoptotic and autophagic signaling in the heart of hyperhomocysteinemic rats

**Fig. 5:**
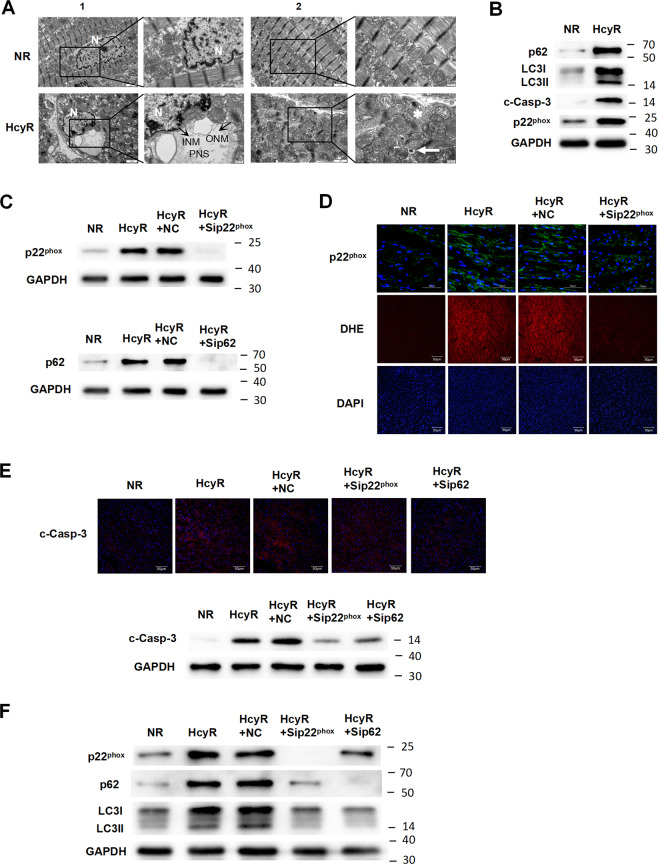
Activation of the NOX–p62-signaling pathway induces autophagy and apoptosis in the heart of hyperhomocysteinemic rats. **A** Ultrastructure of the heart from NR and HcyR. The representative transmission electron microscopic (TEM) images of nuclear [1] and other regions [2] of the left ventricles from NR and HcyR are shown. Right panels of image 1 and 2: Details of the swollen perinuclear space (PNS), inner nuclear membrane (INM, black arrow), outer nuclear membrane (ONM, black arrow), autophagosomes (white arrow) and lysosomes (white star). N nucleus. **B** Expression of LC3, p62, cleaved caspase-3 (c-Casp-3), and p22^phox^ in the heart of HcyR and NR. The representative western blot results are shown, with GAPDH expression as an internal control. **C** Expression of p22^phox^ or p62 in the heart of HcyR with sip22^phox^ or sip62 administration. The representative western blot results are shown, with GAPDH expression as an internal control. **D** The representative image of p22^phox^ or dihydroethidium (DHE) staining of the heart of HcyR with Sip22^phox^ administration. **E** Effect of injection of NC, sip22^phox^, or sip62 on the expression of cleaved caspase-3 (c-Casp-3) in the heart of HcyR. The upper panel is the representative image of the cleaved caspase-3 (c-Casp-3) staining, and the lower panel is the representative western blot results, with GAPDH expression as an internal control. **F** Effect of injection of NC, sip22^phox^, or sip62 on the expression of p22^phox^, p62, and LC3. *n* = 7 rats/group. NR normal rat, HcyR homocysteinemic rat, HcyR+NC homocysteinemic rat treated with non-targeting control siRNA, HcyR+Sip22^phox^ homocysteinemic rat treated with p22^phox^ siRNA, HcyR+Sip62 homocysteinemic rat treated with p62 siRNA, c-Casp-3 cleaved Caspase-3.

Since the Cu already exists in vivo, we further investigated the role of the NOX–p62 axis in apoptotic and autophagic signaling in the heart of a hyperhomocysteinemic rat model. We generated a chronic hyperhomocysteinemic rat model based on our previous study [25] (HcyR, Fig. S4). Figure S5 showed that Hcy administration does not cause malnutrition in the animals. However, the heart of HcyR exhibited significant histological changes. Compared with the control, the ultrastructure of the left ventricle from HcyR exhibited the characteristic features of autosis (swollen PNS, focal separation of INM and ONM, and abundant lysosomes and autophagosomes, Fig. 5A). Consistent with the results of in vitro studies, LC3-II conversion, the expression of p22^phox^ and p62, as well as the cleaved caspase-3 were increased in the heart from HcyR (Fig. 5B). Since gene knockdown in rats with a simple RNAi strategy has been proven to be a feasible method [26], we treated HcyR with Sip22^phox^ or Sip62 to investigate the role of NOX–p62 axis in the heart of HcyR. As shown in Fig. 5C, RNAi administration significantly diminished the upregulation of p22^phox^ and p62 in the heart from HcyR. ROS generation was detected by DHE staining. Hearts from HcyR showed a marked increase in total ROS production, whereas Sip22^phox^ administration decreased p22^phox^ expression and greatly inhibited ROS accumulation in hearts from HcyR (Fig. 5D), indicating that the ROS generation in the heart of HcyR is p22^phox^-dependent. Using immunofluorescence and western blot analysis, we found the cleaved caspase-3 levels were significantly increased in the heart of HcyR, whereas the knockdown of p22^phox^ or p62 significantly diminished the amount of cleaved caspase-3 (Fig. 5E). Meanwhile, Sip22^phox^ administration diminished the expression of p62, and the knockdown of p22^phox^ or p62 in vivo decreased the LC3-II conversion in the heart of HcyR (Fig. 5F). Altogether, these results suggest that the NOX–p62 axis regulates both apoptotic and autophagic signaling in the heart of HcyR.

### Knockdown of p22^phox^ or p62 alleviate cardiac dysfunction in hyperhomocysteinemic rats

**Fig. 6:**
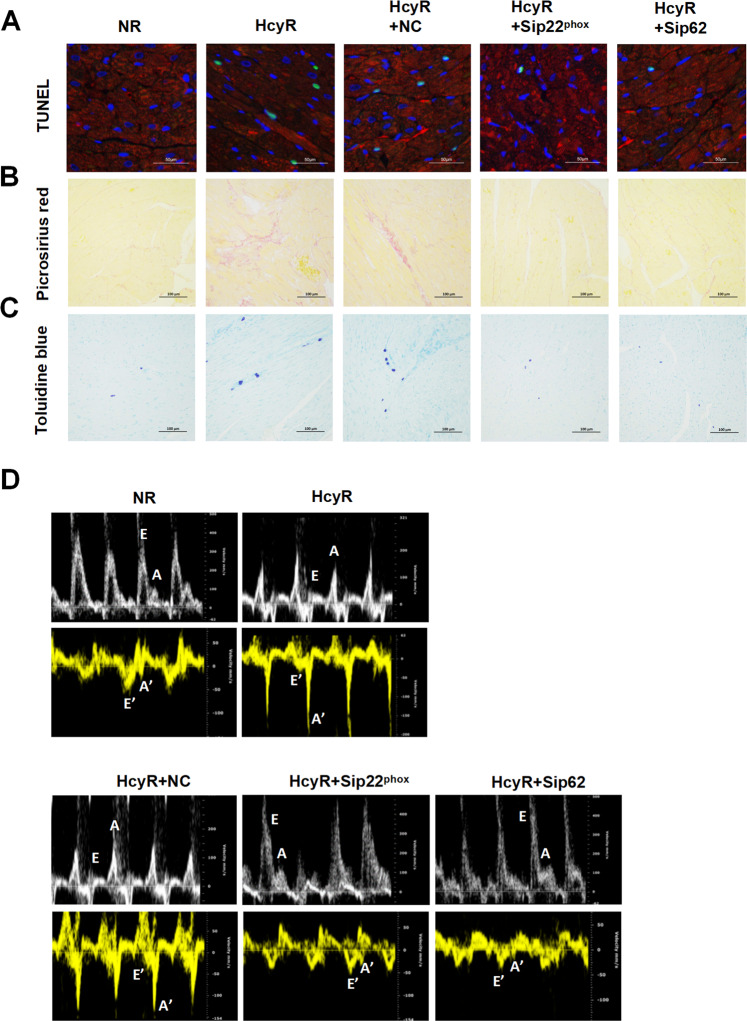
Effect of sip22^phox^ or sip62 administration on cardiac morphology and function in HcyR. The representative myocardial sections show cardiomyocyte cross-sectional area of TUNEL positivity (**A**), myocardial fibrosis (**B**), and mast cell infiltration (**C**). (**D**) Representative transmitral flow velocity and Doppler tissue imaging (DTI) mitral annulus velocity patterns. *E* peak velocity of early mitral inflow, *A* peak velocity of late mitral inflow, *E*′ early diastolic velocity of the mitral annulus, *A*′ late diastolic mitral annulus velocity. *n* = 7 rats/group. NR: HcyR+NC, HcyR+Sip22^phox^, HcyR+Sip62 (see Fig. 5).

We further investigated the relationship between NOX–p62 axis-mediated cardiomyocyte damage and the development of cardiac dysfunction in HcyR by morphometric measurement and echocardiographic analysis. Compared with NR, HcyR had a significant increase in the number of TUNEL-positive cardiomyocytes, fibrotic replacement and mast cells infiltration (Fig. 6A–C). By comparison, Sip22^phox^ or Sip62 administration reduced the TUNEL positivity, myocardial collagen accumulation and mast cell infiltration (Fig. 6A–C). Table 1 shows that the difference reached statistical significance. Furthermore, cardiac function was examined by echocardiography in HcyR with or without Sip22^phox^ or Sip62 administration. By M-mode echocardiogram, we found no significant changes in baseline systolic function in all groups (Fig. S6 and Table 2), suggesting that the NOX–p62 axis does not affect cardiac systolic function in HcyR. However, the Doppler flow and Doppler tissue imaging showed that the *E*/*A* velocity ratio, an estimate of LV diastolic function, exhibited an inversed pattern in HcyR (Fig. 6D, Table 2), suggesting a significant abnormality in diastolic function in HcyR. This was in agreement with previous reports that hyperhomocysteinemia leads to diastolic dysfunction of the rats [27]. *E*/*E*′, another index to evaluate diastolic dysfunction, was not increased in HcyR (Table 2), which might be due to a weaker correlation between *E*/*E*′ and filling pressures in those with normal ejection fraction, as reported by other studies [28, 29]. Importantly, Sip22^phox^ or Sip62 administration prevented the LV diastolic dysfunction and restored the normal velocity patterns in HcyR (Fig. 6D, Table 2), indicating that the NOX–p62 axis is essential for diastolic dysfunction in HcyR.

**Table 1:** Effect of sip22^phox^ or sip62 administration on myocardial fibrosis and mast cell infiltration in the left ventricle from HcyR.

Parameter	NR (*n* = 7)	HcyR (*n* = 7)	HcyR+NC (*n* = 7)	HcyR+Sip22^phox^ (*n* = 7)	HcyR+Sip62 (*n* = 7)
Collagen%	0.21 ± 0.14	1.64 ± 1.14 ^**^	1.53 ± 0.85	0.38 ± 0.30 ^##^	0.44 ± 0.10 ^&&^
Total mast cell/section	12 ± 3	27 ± 8 ^***^	25 ± 7	16 ± 6 ^#^	15 ± 5 ^&^

Values are means ± SD; *n* = no. of rats/group. Collagen% is the ratio of collagen to luminal area.

**p < 0.01, ***p < 0.001 vs. NR; ^#^*p* < 0.05, ^##^*p* < 0.01 vs. HcyR+NC; ^&^*p* < 0.05, ^&&^*p* < 0.01 vs. HcyR+NC.

*NR* normal rat, HcyR homocysteinemic rat, HcyR+NC homocysteinemic rat treated with non-targeting control siRNA, HcyR+Sip22phox homocysteinemic rat treated with p22phox siRNA, HcyR+Sip62 homocysteinemic rat treated with p62 siRNA.

**Table 2:** Echocardiographic data.

	NR (*n* = 7)	HcyR (*n* = 7)	HcyR+NC (*n* = 7)	HcyR+Sip22^phox^ (*n* = 6)	HcyR+Sip62 (*n* = 6)
Heart rate, beats/min	479 ± 44	485 ± 33	500 ± 26	487 ± 28	494 ± 22
LVEDD (mm)	5.56 ± 0.12	5.47 ± 0.43	5.56 ± 0.32	5.55 ± 0.26	5.50 ± 0.19
LVEF%	90.25 ± 2.14	89.36 ± 4.29	90.34 ± 2.01	90.23 ± 2.13	90.03 ± 2.46
*E* (mm/s)	326.70 ± 25.20	81.64 ± 71.88^***^	74.82 ± 81.05	266.50 ± 82.85 ^##^	303.40 ± 69.17^§§§^
*A* (mm/s)	115.80 ± 11.08	137.40 ± 16.61^*^	140.50 ± 15.27	153.30 ± 41.63	157.20 ± 42.83
*E*/*A*	2.85 ± 0.41	0.58 ± 0.0.46^***^	0.56 ± 0.67	1.93 ± 1.085^##^	2.03 ± 0.71^§§§^
*E’* (mm/s)	49.43 ± 4.94	37.35 ± 4.47^***^	40.30 ± 6.01	48.34 ± 6.18^#^	49.74 ± 2.28^§§^
*A*′ (mm/s)	31.54 ± 3.69	105.70 ± 28.40 ^***^	102.0 ± 31.06	35.43 ± 8.16^###^	35.57 ± 8.29^§§§^
*E*′/*A*′	1.58 ± 0.23	0.40 ± 0.20^***^	0.47 ± 0.31	1.42 ± 0.53^##^	1.46 ± 0.35^§§§^
*E*/*E*′	6.65 ± 0.69	2.17 ± 1.89	1.89 ± 2.02	5.73 ± 1.51	6.07 ± 1.21

Data are mean ± SD. *n* = no. of rats/group that were successfully evaluated by Doppler imaging.

*LVEDD* LV end-diastolic diameter, *LVEF* left ventricular ejection fraction, *E* peak velocity of early mitral inflow, *A* peak velocity of late mitral inflow, *E*′ early diastolic velocity of the mitral annulus, *A*′ late diastolic mitral annulus velocity.

*p < 0.05 vs. NR; ***p < 0.001 vs. NR; ^#^*p* < 0.05 vs. HcyR+NC; ^##^*p* < 0.01 vs. HcyR+NC; ^###^*p* < 0.001 vs. HcyR+NC; ^§§^*p* < 0.01 vs. HcyR+NC; ^§§§^*p* < 0.001 vs. HcyR+NC. NR, HcyR+NC, HcyR+Sip22^phox^, HcyR+Sip62: see Table 1.

## Discussion

The association between hyperhomocysteinemia with atherosclerosis has been long established by McCully in 1969 [30]. In addition to the vascular effects, Hcy has been associated with clinically cardiac dysfunction [31], indicating that Hcy may also act on cardiomyocytes to induce myocardial remodeling. Importantly, high levels of Hcy associated with cardiovascular events are accompanied by increased Cu concentrations in the blood [32, 33]. However, the role of Cu and the exact mechanisms are largely unknown. The present study provided evidence to demonstrate that the combination of Hcy and Cu^2+^ induces the crosstalk of apoptotic and autophagic cell death in cardiomyocytes. This is operated through the p22^phox^ activation and NOX-mediated p62 upregulation both ex vivo and in vivo of hyperhomocysteinemic rats. Importantly, the NOX–p62 axis-mediated apoptotic and autophagic signaling induces cardiac dysfunction in hyperhomocysteinemic rats, suggesting a previously unidentified mechanism for Hcy-mediated cardiac dysfunction.

Damaged cardiomyocytes show characteristics of autophagy during heart failure [34]. However, it remains unclear whether autophagy is a cell survival response or a death execution mechanism in the failed cardiomyocytes. Recently, a novel form of Na^+^, K^+^-ATPase-dependent autophagic cell death, termed autosis, is identified, which is triggered by high levels of cellular autophagy [5]. To date, only autophagy-inducing peptides, starvation and cerebral hypoxia-ischemia are demonstrated to induce autosis, the upstream signaling pathways and the relationship between autosis and other forms of cell death are largely unknown. Our findings support the notion that excessive accumulation of p62/SQSTM1 in cardiomyocytes with Hcy and Cu^2+^ treatment triggers both autosis and apoptosis. Unlike other autophagy adaptors, p62/SQSTM1 is a central hub due to its ability to interact with the key signaling proteins through its abundant protein-interacting sequences, and functions at a critical decision point to control cell death or survival [17]. To date, the understanding on the function of p62 remains incomplete, and the change and pathophysiological role of p62 in cardiomyocytes remain largely unknown. Studies have shown that p62 expression is significantly increased in mouse proteinopathic hearts that promotes aggresome formation and autophagy activation, and protects cardiomyocytes against proteotoxic stress [17]. On the contrary, our study demonstrated that p62 accumulates in cardiomyocytes with Hcy and Cu^2+^ treatment and in the heart of hyperhomocysteinemic rats, and plays an important role in both autosis and apoptosis in cardiomyocytes.

Although autophagy and apoptosis are markedly different processes, several pathways regulate both autophagic and apoptotic machinery, and may induce the crosstalk between them. The current knowledge on the relationship between apoptotic and autophagic death is controversial, especially, the relationship of apoptosis and autosis is rarely reported. They may cooperate, coexist, or antagonize each other. The interaction of Bcl-2 and beclin 1, the cleavage of beclin 1 by caspase and the depleting endogenous inhibitors of apoptosis may play important roles in the crosstalk [35]. In our study, we found that there is crosstalk between Hcy and Cu^2+^-induced apoptosis and autosis of cardiomyocyte, and the Bcl-2 degradation might play a role in the physiologic link, which needs further investigation.

ROS has been shown to serve as an important intermediate factor in the correlation between Hcy and endothelial dysfunction [36]. Our recent study also has suggested that the effect of Hcy on endothelium occurs via oxidative stress in vitro and in vivo of hyperhomocysteinemic rats [25]. ROS participates in the interplay between autophagy and apoptosis by its ability to mediate the redox signaling pathways. NOX is one of the major sources for cellular ROS whose activation occurs either through translocation of regulatory subunits and post-translational protein modification, or through an increased expression of oxidase subunit. In the present study, we found the expression of p22^phox^ is upregulated in cardiomyocytes in the presence of Hcy and Cu^2+^ both in vitro and in vivo, and the p22^phox^ RNAi experiments showed that ROS production is a consequence of a p22^phox^-dependent NOX activation that regulates the interaction of autophagy and apoptosis.

A positive correlation between high plasma Hcy concentrations and an increased risk of left ventricular hypertrophy was observed in patients with end-stage renal disease [31]. Hcy administration is shown to lead to pathological ventricular hypertrophy in normotensive rats [27], and to exacerbate adverse cardiac remodeling and diastolic dysfunction in hypertensive rats [37]. However, the site and cellular mechanisms underlying the adverse cardiac remodeling induced by Hcy are largely unknown. Moreover, Hcy-lowering therapies fail to reduce the risk of cardiovascular diseases despite a substantial reduction in total Hcy levels with vitamin treatment [38]. Our study proved that Hcy combined with Cu^2+^ act on cardiomyocytes directly in the absence of endothelial cells, previously believed to play the central role in the Hcy-induced cardiovascular disease, and thus lead to cardiac dysfunction.

This study shed new light on the understanding of hyperhomocysteinemia-induced cardiac dysfunction. We demonstrate the combination of Hcy and Cu^2+^ induces both apoptosis and autosis of cardiomyocytes, which involves the NOX-originated ROS generation, followed by the p62 upregulation and cell death. Importantly, interrupting the NOX–p62 axis prevents diastolic dysfunction in HcyR. This may lead to the development of new strategies to treat hyperhomocysteinemia-mediated cardiovascular diseases.

## Materials and methods

### Reagents

Homocysteine, copper (II) chloride dehydrate, chloroquine (CQ), VAS2870, zVADfmk, propidium iodide (PI), Digoxin, Cytotoxicity detection kit and GAPDH (cat. no. G8795) were purchased from Sigma-Aldrich (St. Louis, MO, USA). CM-H2DCF-DA, acridine orange, dihydroethidium, and Mitotracker red were purchased from Thermo Fisher Scientific (Waltham, MA, USA). The Annexin V-fluorescein isothiocyanate (FITC) and caspase-3 activity assay kit were purchased from BD Biosciences (San Jose, CA, USA). Cytochrome c release assay kit was from EMD Biosciences-Calbiochem (San Diego, CA, USA). TransIT was from Mirus Bio (Madison, WI, USA). Atg7 siRNA, p62 siRNA, p22^phox^ siRNA and Lipofectamine RNAiMAX were purchased from Thermo Fisher Scientific (Waltham, MA, USA). Anti-cleaved caspase-3 (cat. no. 9664), anti-p62 (cat. no. 23214), anti-p22^phox^ (cat. no. 27297), and anti-Atg7 (cat. no. 8558) were from Cell Signaling Technology (Danvers, MA, USA). Anti-LC3A (cat. no. NB 10-2331) was from NOVUS Biologicals (Littleton, CO, USA). Anti-PARP-1 (cat. no. 13371-1-AP) and anti-Beclin 1 (cat. no. 11306-1-AP) was from Protintech (Wuhan, China). Anti-p22^phox^ (cat. no. sc-271968) and anti-Bcl-2 (cat. no. sc-7382) were from Santa Cruz Biotechnology, Inc. (Santa Cruz, CA, USA).

### Cell culture

Primary cultures of neonatal ventricular myocytes from 2-day-old Sprague-Dawley rats were prepared as previously described. In brief, ventricles were digested with collagenase (1 mg/ml) and pancreatin (1 mg/ml). Cells were recovered by centrifugation (5 min, 1000 rpm), resuspended in plating medium (85% DMEM, 15% FBS, 100 U/ml of penicillin and streptomycin) and pre-plated on 60-mm culture dishes for 2 h to remove non-myocytes. The non-adherent cardiomyocytes were plated at 1 × 10^6^ cells per dish. After 24 h, cardiomyocytes were washed and cultured for another 3 days prior use. H9c2 cells, a clonal line derived from rat heart (National Collection of Authenticated Cell Cultures, China), was maintained in DMEM medium supplemented with 10% fetal bovine serum (FBS) at 37 °C in a humidified incubator with 5% CO_2_. The H9c2 cells were authenticated by STR profiling and tested for mycoplasma contamination.

### Animals and treatment

Male 3-month-old Sprague-Dawley (SD) rats from the Animal Center, Jiangxi University of Traditional Chinese Medicine, PR China were used for the experiments. The animals were maintained under normal laboratory condition of humidity (50 ± 10%), temperature (25 ± 2 °C) and a 12-h light/dark cycle for 7 days, and allowed free access to food and water ad libitum. The protocol complied with the guidelines of Nanchang University, PR China for the care and use of laboratory animals. Chronic hyperhomocysteinemia was induced as described previously [25]. Briefly, Hcy dissolved in 0.9% NaCl was buffered to pH 7.4, and administering subcutaneously twice a day at 8 h intervals from day 1 to day 21 of male SD rats. During the first week, rats receive 0.3 μmol Hcy/g body weight, and 0.4 and 0.6 μmol Hcy/g body weight during the second and third weeks, respectively. Rats subjected to this treatment achieved plasma Hcy levels similar to those found in homocystinuric patients (Fig. S4). For p62 and p22^phox^ small-interfering RNA (siRNA) treatment, 100 μg of siRNA (or a control siRNA) was administered by intravenous injection on day 5 and day 10 of Hcy injection and 50 μg on day 15. The siRNA was complexed with a polymer from TransIT in vivo gene delivery system and delivered according to the manufacturer’s instructions. The rats were randomized into the above groups. The protocol of the study was designed according to our pilot studies and previous reports that had used in vivo siRNA successfully to target genes in rats [39–41].

### siRNA transfection

siRNA targeting Atg7, p62, p22^phox^, or non-targeting sequence control siRNA (NC) was transfected to H9c2 cells with Lipofectamine RNAiMAX (Thermo Fisher Scientific) according to the manufacturer’s instructions. The transfected H9c2 cells were washed with PBS, and then incubated in new culture media for additional time for Hcy and CuCl_2_ treatment, followed by AVOs detection, western blot and cell viability analysis as indicated in the figure legends.

### Cell viability assays

Cell death was assessed by the measurement of lactate dehydrogenase (LDH), released from dead cells or determined by flow cytometry (FACS-Calibur, BD Biosciences, CA) after double staining cells with annexin V-FITC and PI as described previously [42] or after staining cells with Mitotracker red. Neonatal cardiomyocytes or H9c2 cells were treated with various compounds under the conditions indicated in the figure legends. LDH release into culture supernatants was detected by colorimetric enzyme-linked immunosorbent assay, using the cytotoxicity detection kit (LDH) from Sigma, according to manufacturers’ instructions. All assays were carried out at least three times.

### Analysis of mitochondrial cytochrome c release and caspase-3 activation

A cytochrome c release kit (EMD Millipore, San Diego, CA, USA) and caspase-3 activation assay kit (BD Biosciences, San Jose, CA, USA) were used to measure the loss of mitochondrial cytochrome c and the levels of activated caspase-3, according to the manufacturer’s instructions.

### Western blot analysis

After being cultured under various experimental conditions, the cells were harvested and washed in cold PBS, and directly solubilized in buffered solution containing 10 mM pH 7.6 Tris–HCl, 1% SDS and the complete protease inhibitor cocktail (Roche Diagnostics Ltd, Mannheim, Germany). Frozen ventricle was homogenized in ice-cold lysis buffer containing 50 mM pH 7.4 Tris–HCl, 150 mM NaCl, 1 mM EDTA, 1% Triton X-100, 0.5% Na-deoxycholate, 0.1% SDS, 1 mM PMSF, 1 mg/ml aprotinin, leupeptin and pepstatin, 1 mM Na_3_VO_4_, 1 mM NaF. The total protein concentrations were quantified using a BCA Protein Assay Kit (Pierce Biotechnology, #23225), and then adjusted to 2 μg/mL with sample buffer containing 250 mM pH 6.8 Tris–HCl, 4% SDS, 10% glycerol, 0.006% bromophenol blue, and 2% β-mercaptoethanol. The lysates were heated at 95 °C for 10 min, and equal amounts of proteins were separated on SDS–PAGE in a Mini-Protean II Dual Slab Cell (Bio-Rad Laboratories, Hercules, CA, USA). The proteins were then transferred on to nitrocellulose membranes using a Mini Trans-Blot Transfer Cell (Bio-Rad Laboratories). The transfer was performed at 4 °C for 2 h at a constant voltage setting of 110 V. The blots were blocked in 5% skimmed milk for 1 h at room temperature. The membranes were then probed with the following primary antibodies: PARP-1, cleaved caspase-3, LC3, p62, p22^phox^, Atg7, Bcl-2 and beclin 1, all at 1:1000 dilution, and GAPDH at 1:10,000 dilution. After incubation for 2 h at room temperature, the blots were washed three times for 10 min in PBS containing 0.1% Tween-20, and then incubated for 1 h at room temperature in the following secondary antibodies: goat anti-rabbit polyclonal antibody for PARP-1, cleaved caspase-3, LC3, p62, p22^phox^, Atg7 and beclin 1 detection, all at 1:3000 dilution, goat anti-mouse polyclonal antibody for Bcl-2 and GAPDH detection at 1:3000 and 1:10,000 dilution, respectively. The blots were then washed three times for 10 min with the same buffer as above and incubated in enhanced chemiluminescence detection reagents (GE Healthcare Life Sciences, Chalfont, UK) for 1 min. The blots were then exposed to an X-OMAT AR X-ray film (Kodak, Rochester, NY, USA) for between 10 s and 5 min.

### Transmission electron microscopic investigation

Neonatal cardiomyocytes were treated with 800 μM Hcy and 20 μM CuCl_2_ for 12 and 20 h, respectively. A small piece of the left ventricle was removed from the rat immediately after decapitation, placed in fixative, and minced into 3–4 mm rings. The Cells and tissues were fixed in 2.5% glutaraldehyde in 0.1 M cacodylate buffer (pH 7.4) at 4 °C for 3 h, then rinsed in buffer, postfixed in 1% OsO_4_, dehydrated in ethanol and embedded in Epon 812. Thin sections after staining with 2% uranyl acetate and lead citrate were examined with TECNAI G2 20 TWIN (FEI, Eindhoven, The Netherlands).

### Detection and quantification of acidic vesicular organelles (AVOs) with acridine orange

Detection and ratiometric analysis of acridine orange (AO) staining of autophagy was performed as previously described [23]. Autophagy is characterized by the formation of acidic vesicular organelles (AVOs). AO is a marker of AVOs that fluoresces green in the whole cell except in acidic compartments like late autophagosomes, where it fluoresces red [23]. The cells were cultured under various experimental conditions, and then incubated with 1 μg/ml AO for 15 min at room temperature in the dark, followed by visualization with a confocal laser scanning microscope Zeiss LSM 700. To quantify the percentage of cells with AVOs (red to green fluorescence intensity ratio—R/GFIR), the AO-stained samples were measured using flow cytometry (FACS-Calibur, BD Biosciences, CA), and the results were analyzed according to a red to green fluorescence intensity ratio (R/GFIR)-based threshold using the built-in software.

### Detection of cellular ROS levels

The cellular ROS levels were detected with a fluorescent probe of CM-H2DCF-DA. In brief, the cells were cultured under various experimental conditions, and then incubated with 1 μM CM-H2DCF-DA for 60 min at 37 °C in the dark. After washing twice, the resulting samples were measured using flow cytometry (FACS-Calibur, BD Biosciences, CA), and the results were analyzed based on forward scatter/side scatter gating to differentiate between dead and viable cells using the built-in software.

### Assay for cellular NADPH oxidase activity

Validation of these assays was reported previously [43]. Briefly, 5–7 μg of the neonatal cardiomyocyte homogenate fraction were transferred to scintillation vials in phosphate buffer (50 mM, pH 7.0) containing 150 mM NaCl and 1 mM EGTA, followed by an addition of 5 μM lucigenin in dark. The chemiluminescence was recorded every minute for 15 min by a liquid scintillation counter (Wallac 1409, Turku, Finland) switched to the out-of-coincidence mode. The background was subtracted from total count.

### Myocardial histomorphometric analysis

Immunofluorescent imaging of heart tissue was performed using standard techniques. Briefly, the fresh heart tissues were embedded in OCT compound and sectioned 5 μm thick in a cryostat (Leica CM3050S-III). Sections were air-dried and stored at −80 °C until used. After removal of the O.C.T. compound by washing with water, sections were permeabilized with 3% Triton X-100 for 30 min at room temperature. After washing with PBS three times for 5 min each at room temperature, sections were blocked with 5% BSA for 1 h at room temperature. Sections were then incubated with primary antibodies to p22^phox^ (sc-271968, Santa Cruz) and Cleaved Caspase-3 (9664, Cell Signaling Technology) diluted 200-fold in 5% BSA overnight at 4 °C. Secondary antibodies, Alexa 488- and 594-conjugated anti-mouse and rabbit IgG (ab150117, ab150080, Abcam), respectively, were used at a 200-fold dilution in 5% BSA for 1 h at room temperature. Nuclei were counterstained with DAPI (0100-20, SouthernBiotech). Fluorescent images were captured in the Olympus BX53 (Tokyo, Japan) fluorescent microscope. For the picrosirius red and toluidine blue staining, the fresh heart tissues were fixed in cold 4% formaldehyde for more than 24 h, processing, and embedding in paraffin, 5 μm-thick slices were obtained that were sectioned at equally spaced intervals, and stained with picrosirius red or toluidine blue to identify fibrillar collagen and mast cells, respectively. The samples were imaged with an Olympus BX51 microscope (Tokyo, Japan), and collagen area was calculated as percentage of total LV myocardial area with the use of Image-Pro Plus 6.0. TUNEL staining used the DeadEnd fluorometric TUNEL system (Promega), according to the manufacturer’s instructions, counterstained with α-cardiac actin (PA5-21396, Invitrogen) and DAPI (0100-20, SouthernBiotech) and imaged with the use of a Nikon Eclipse Ci-L microscope (Tokyo, Japan).

### In situ detection of cardiac ROS production

ROS generation was measured by detection of fluorescent dihidroethidium (DHE) oxidation products as described [25]. Briefly, unfixed heart were cryopreserved by incubation with PBS containing 30% sucrose for 1–2 h, included in OCT, frozen, and 10 μm cross-sections were obtained in a Leica CM1850 cryostat (Leica, Germany). Sections were incubated in a humidified chamber at 37 °C for 30 min in HEPES-buffered solution containing 130 mM NaCl, 5 mM KCl, 1.2 mM MgCl_2_, 10 mM glucose, 10 mM HEPES, buffered to pH 7.3. Then the sections were further incubated for 30 min in HEPES solutions containing DHE in the dark. Images were obtained with an Olympus BX51 microscope (Tokyo, Japan). This fluorescence was evaluated in at least three sections of each preparation.

### Echocardiography

Transthoracic two-dimensional M-mode echocardiogram, pulsed wave Doppler spectral tracings and tissue Doppler imaging were obtained using a Vevo 2100 pulsed doppler spectrum tracking ultrasound system (VisualSonics, Toronto, Canada) with a 15-MHz transducer. The rats were anesthetized with pentobarbital sodium (50 mg/kg). M-mode tracings were used to measure left ventricular (LV) wall thickness and LV end-diastolic diameter (LVEDD). Color Doppler was used to show accurately the mitral valve inflow and to obtain a sharper signal from the early ventricular filling peak velocity (*E* wave) and late filling velocity (*A* wave). Pulse tissue Doppler imaging was used to measure the early peak diastolic velocity (*E*′) and the late peak diastolic velocity (*A*′). All examinations were performed by the same personnel.

### Statistical analysis

All experiments were performed at least three times. Statistical significance was estimated by using Student’s *t*-test. *p* < 0.05 was considered to possess a statistically significant difference. Plots were produced on GraphPad Prism 8 software (GraphPadSoftware, Inc., La Jolla, CA, USA).

## Supplementary information

Supplementary figure 1-6

## Data Availability

The data that support the findings of this study are available from the corresponding author upon reasonable request.
