# Supplementary material for: Induction of apoptosis and autosis in cardiomyocytes by the combination of homocysteine and copper via NOX-mediated p62 expression
Source: Cell Death Discov. 2022 Feb 21;8:75. doi: 10.1038/s41420-022-00870-4 (PMC8860999; doi:10.1038/s41420-022-00870-4)
Supplement: Supplementary file 1 — Supplementary figure 1-6 [file 41420_2022_870_MOESM1_ESM.pdf]

Supplementary information contains the supplementary Figure 1-6 of the manuscript.

Figure S1

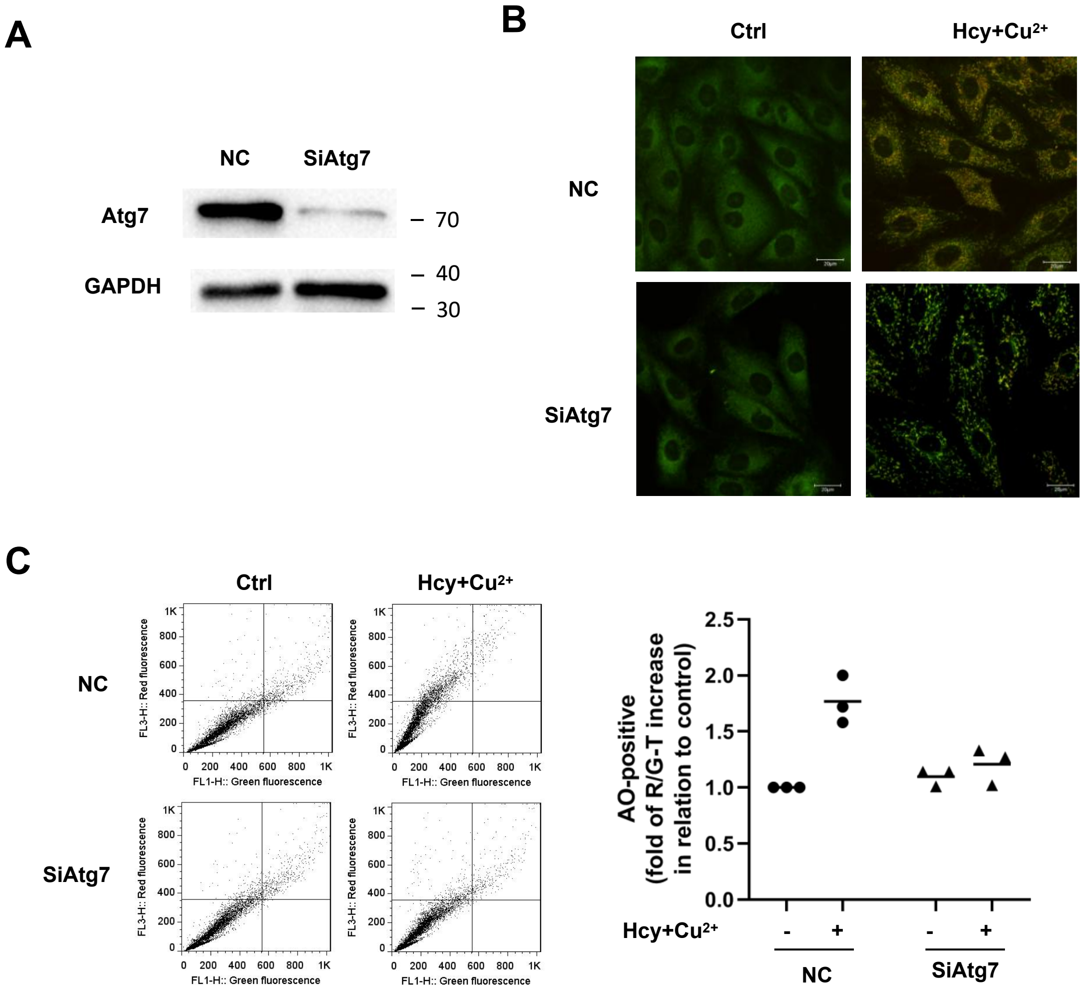

Figure S1. Atg7 knockdown attenuates Hcy and Cu^{2+} induced autophagic activity in cardiomyocytes. (A) Knockdown efficiency of Atg7 in cardiomyocytes. H9c2 cells were infected with Atg7 siRNA or non-targeting siRNA control (NC), and cell lysates were assayed for Atg7 using western blot analysis. **(B)** Reduced AVOs development in H9c2 against Hcy and Cu^{2+} treatment by Atg7 knockdown. H9c2 cells infected with Atg7 siRNA or non-targeting siRNA control (NC) was treated with Hcy and $CuCl_2$ for 12 hours. Representative images of acridine orange-stained H9c2 cells are shown. **(C)** Quantification of acridine orange staining using flow cytometry. Representative dot plots of three separate experiments are shown (left panel). The bar (right panel) represents the mean of three separate experiments, each measured the proportion of the events above the threshold with R/GFIR-T. All the experiments above were performed three times. SiAtg7: Atg7 siRNA; Ctrl, NC, CQ, R/GFIR: See legends of **Figure 1, 3**.

Figure S2

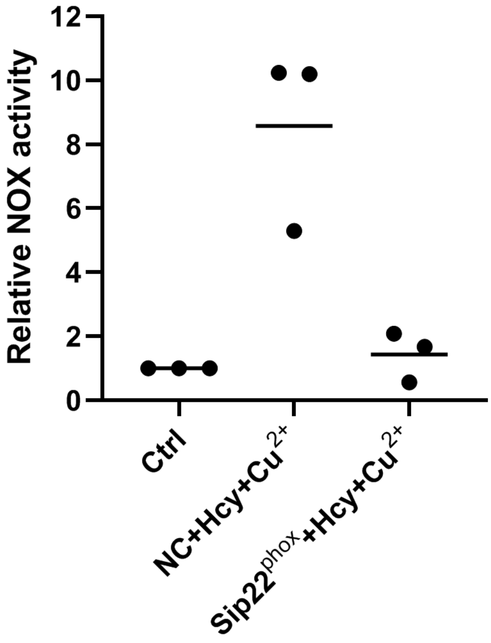

Figure S2. Knockdown of p22phox attenuates Hcy and Cu^{2+} induced NOX activity in cardiomyocytes. H9c2 cells infected with p22phox siRNA or non-targeting siRNA control (NC) was treated with Hcy and $CuCl_2$ for 8 hours. NOX activity was determined by chemiluminescence assays. Each bar represents the mean of three separate experiments. Ctrl, NOX, NC, Sip22phox: See legend of **Figure 5**.

Figure S3

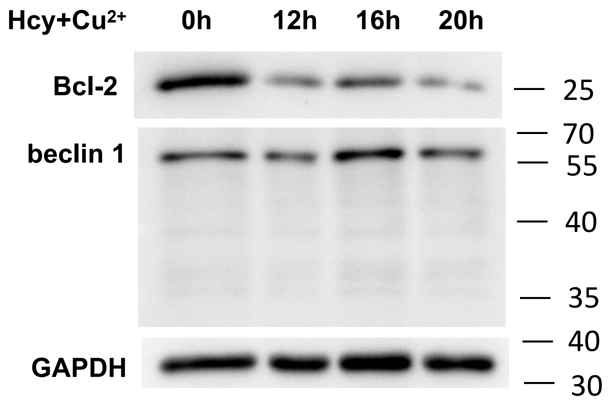

Figure S3. Expression of Bcl-2 and beclin 1 in cardiomyocyte with Hcy and Cu^{2+} incubation. H9c2 cells were treated with 800 μM Hcy and 20 μM $CuCl_2$. Expression of Bcl-2 and beclin 1 at given time points was assessed by western blot analysis. The representative western blot results are shown, with GAPDH expression as an internal control. The experiments were performed three times.

Figure S4

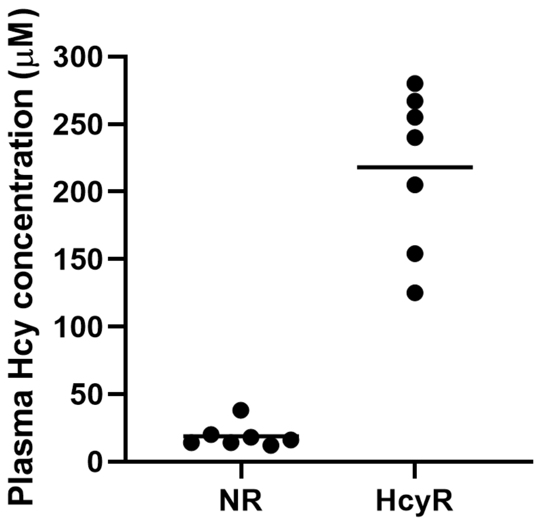

Figure S4. Hcy plasma levels with or without Hcy administration. The bar represents the mean of each group. n=7 rats/group. NR: Normal rat; HcyR: Homocysteinemic rat

Figure S5

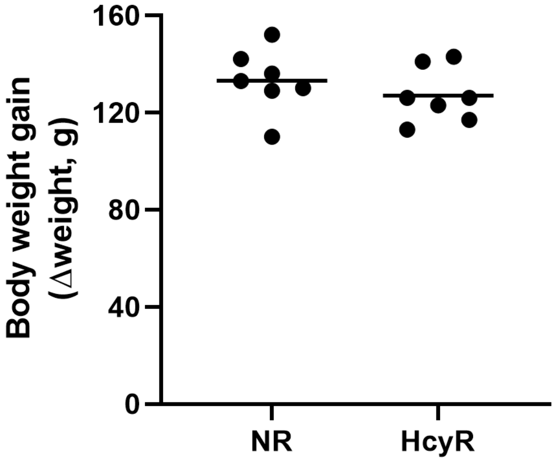

Figure S5. Effect of Hcy on body weight gain in rat. n=7 rats/group.
NR, HcyR: See legend of **Figure S5**.

Figure S6

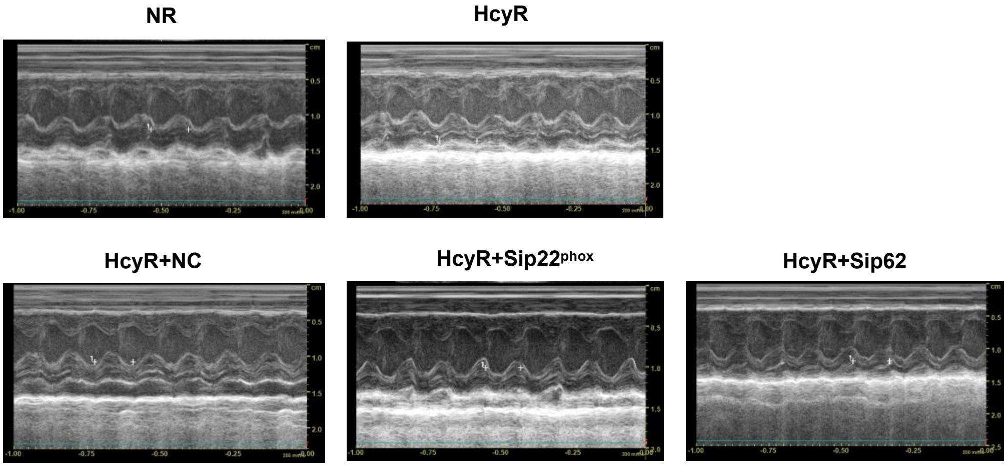

Figure S6. Echocardiographic assessment of cardiac function. Representative images of M-mode echocardiography are shown. n=7 rats/group. NR: Normal rat; HcyR: Homocysteinemic rat; HcyR+NC: Homocysteinemic rat treated with non-targeting control siRNA; HcyR+Sip22^{phox}: Homocysteinemic rat treated with p22^{phox} siRNA; HcyR+Sip62: Homocysteinemic rat treated with p62 siRNA.
